# Gene-based anticoagulation regimens for an infant after mitral-valve replacement: A case report

**DOI:** 10.1097/MD.0000000000018651

**Published:** 2020-01-10

**Authors:** Hua Cao, Xiaotong Xia, Jinglan Fu, Tingting Wu, Wenjun Chen, Ying Dai, Xuan Xia, Jinhua Zhang

**Affiliations:** a Department of Pharmacy, Fujian Medical University Union Hospital; b College of pharmacy, Fujian Medical University; c Department of Cardiac Surgery, Fujian Medical University Union Hospital ^d^ Fujian Maternity and Children Health Hospital, Fuzhou, Fujian, PR China.

**Keywords:** anticoagulation, gene, heart valves, infant, online system

## Abstract

**Rationale:**

Heart-valve replacement is one of the main surgical methods for various heart-valve diseases. Warfarin is the only oral anticoagulant used for thrombosis prevention after heart-valve replacement. However, warfarin has a narrow therapeutic window, large differences in efficacy between individuals, and can be affected by drugs, food and disease status.

**Patient concerns:**

We used the Hamberg model to develop an anticoagulation regimen for a 10-month-old Chinese male after mitral-valve replacement.

**Diagnoses:**

Echocardiography revealed mitral malformation with severe regurgitation, patent foramen ovale, thickening of the left ventricular wall, enlargement of the left atrium, and the overall systolic function of the left ventricle was lower than normal.

**Interventions:**

First, the patient was treated with Mitral valvuloplasty plus temporary implantation of a pacing wire. Since this was inadequate, he underwent mitral-valve replacement. Then, we used the Hamberg model to develop an anticoagulation regimen.

**Outcomes:**

After discharge from hospital, the pharmacist provided anticoagulation management for this pediatric patient using an “Online Anticoagulation Clinic” (OAC). Point-of-care testing could be employed by the boy's mother at home to obtain the International Normalized Ratio. His time to response was 89.6% during the 6 months after hospital discharge, and adverse reactions such as bleeding or thrombosis did not occur.

**Lessons:**

This is the first time the Hamberg model has been employed to design anticoagulation therapy for an Asian infant. His anticoagulation therapy may be managed using the OAC.

## 1 Introduction

“Heart-valve disease” refers to structural and/or functional abnormalities in a heart valve.^[1]^ Hemodynamic changes due to valve stenosis or closure insufficiency can lead to atrial/ventricular structural changes and dysfunction and, ultimately, to heart failure.^[2]^

Replacement of a heart valve is one of the main surgical methods for various heart-valve diseases.^[1,2]^ The dissimilarity of a prosthetic heart valve to any tissue in the human body causes activation of the blood-clotting system and formation of a valve thrombus, leading to dysfunction, heart failure, and even sudden death. Therefore, patients undergoing valve replacement need standard anticoagulation therapy.^[3]^

Warfarin is the only oral anticoagulant employed for thrombosis prevention after replacement of a heart valve.^[4]^ However, warfarin has a narrow therapeutic window, large differences in efficacy between individuals, and can be affected by drugs, food, and disease status.^[5]^

With the development of pharmacogenetics research, the relationship between genes and warfarin dosing has been clarified. The warfarin metabolism-related cytochrome P450 2C9 gene (CYP2C9) and the target vitamin K-epoxide reductase gene (VKORC1) explain 40–60% of the difference in warfarin dose between patients.^[6]^

The warfarin package insert created by the US Food and Drug Administration (FDA) recommends that expression of VKORC1 and CYP2C9 should be measured before warfarin therapy to improve anticoagulation effects.^[7]^ There are numerous validated genotype-based warfarin dosing models for adults, such as International Warfarin Pharmacogenetics Consortium (IWPC), Gage model and FDA recommendations.^[7–9]^ However, there are only two models for children of European descent (Hamberg and Biss), and there is no model for warfarin dosing for children of non-European descent.^[10]^ The Hamberg model is based on a Warfarin Dosing Calculator (WDC) using JAVA computing-programming language, which can be downloaded from additional files in the study by Hamberg et al.^[11]^ JAVA must be installed before the WDC can be used. The interface of the WDC is uncomplicated and easy to learn.

We applied the WDC to predict the initial and maintenance doses of warfarin in an infant after replacement of a mitral valve. We wished to explore how to provide anticoagulant management for infants using an “Online Anticoagulation Clinic” (OAC).^[2]^ In this way, we hoped to improve anticoagulation efficacy and reduce the risk of thrombosis and bleeding.

## 2 Case report

A 10-month-old Chinese male with a heart murmur of 3-month duration and cough for 7 days was admitted to our hospital. He was susceptible to recurrent colds, and his weight and growth lagged that of his peers. Physical examination upon hospital admission revealed a weight of 8.5 kg, body temperature of 36.6°C, pulse of 136/minute, respiratory rate of 28/minute, oxygen saturation of 96% and heart rate of 136 bpm.

Echocardiography revealed mitral malformation with severe regurgitation, patent foramen ovale, thickening of the left ventricular wall, enlargement of the left atrium, and the overall systolic function of the left ventricle was lower than normal. Electrocardiography demonstrated a P-wave abnormality, high voltage in the left ventricle, and changes in ST-T waves.

Mitral valvuloplasty plus temporary implantation of a pacing wire was done under emergency local anesthesia on 4 December 2017. Brown urine appeared on postoperative day (POD)3, and mechanical hemolysis was considered. Echocardiography showed severe valvular regurgitation, and repeated avulsion after valvular formation was considered. Mitral-valve replacement was undertaken under emergency general anesthesia on 7 December 2017. Anticoagulation therapy was started on POD5.

**Figure 1:**
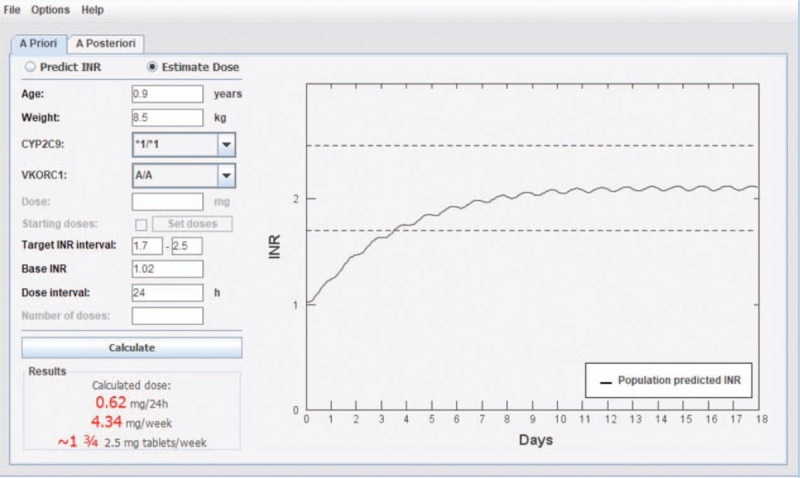
Warfarin Dosing Calculator model-predicted initial dose of warfarin.

The warfarin-related genes in this pediatric patient were CYP2C9∗1/∗1 and VKORC1 AA. We predicted an initial dose of 4.34 mg/week (0.62 mg/24 hour) using the WDC (Fig. 1). However, the WDC is suitable only for children of European descent, and not for Asian children.

After discussion, the final anticoagulant regimen was low-molecular-weight (LMW) heparin (400 AxaIU, q.d.) + warfarin (0.325 mg, q.d.). Three days later, the International Normalized Ratio (INR) was 1.09. Hence, the anticoagulation regimen was changed to LMW heparin (400 AxaIU, q.d.) + warfarin (0.625 mg, q.d.). Two days later, the INR was 1.14. Three days later, the INR was 1.13.

**Figure 2:**
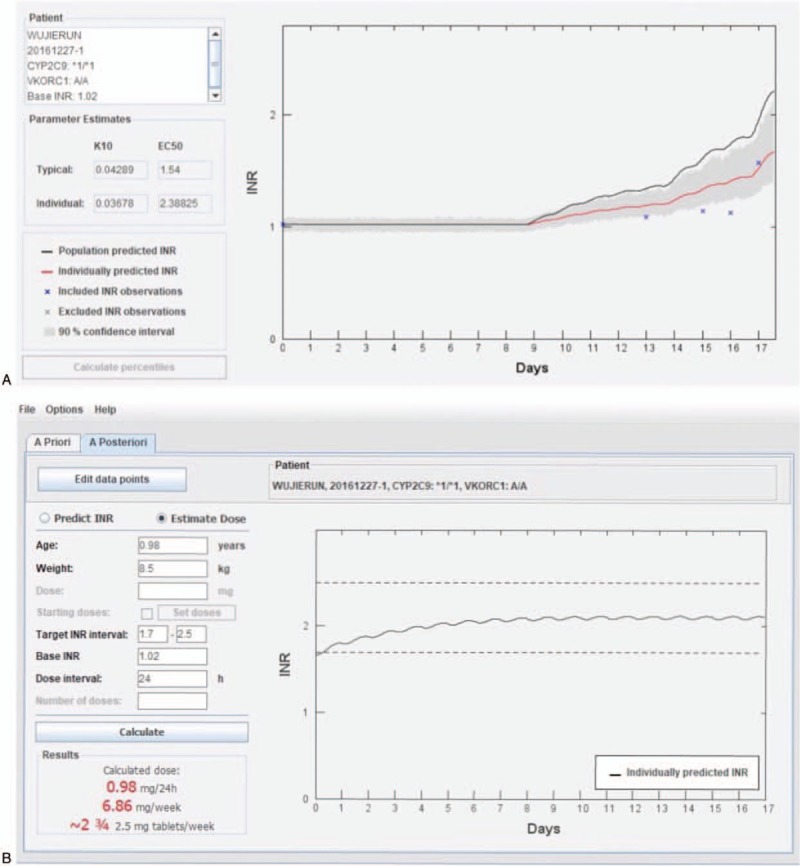
A. WDC model-estimated individual parameters. B. WDC model-calculated maintenance dose. WDC = Warfarin Dosing Calculator.

Using the INR and warfarin-dose data shown above, the WDC was applied again to calculate the maintenance dose of warfarin. The prediction steps were:

(i) importing patient data into the WDC
(ii) estimating the individual model parameters (Fig. 2A)

**Figure 3:**
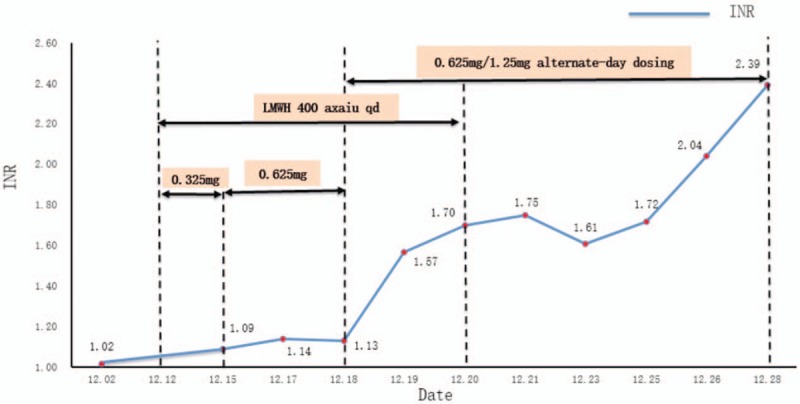
International Normalized Ratio and anticoagulant-drug use of our pediatric patient in hospital.

Finally, the predicted warfarin dose was 6.86 mg/week (0.98 mg/24 hour) with a target INR of 1.7 to 2.5 (Fig. 2B). The dose of each warfarin tablet in China is 2.5 mg, so the anticoagulation regimen was changed to LMW heparin (400 AxaIU, q.d.) + warfarin (0.625 mg, q.d., and 1.25 mg, q.d., at alternate intervals). Two days later, the INR was 1.70, so the target INR had been reached. The next day, LMW heparin was withdrawn, bur warfarin (0.625 mg, q.d., and 1.25 mg, q.d., at alternate intervals) was continued. Before discharge from hospital, the INR remained within the target range (Fig. 3).

“Point-of-care testing” (POCT) refers to use of a portable analytical instrument at the patient's bedside that is easy to operate and enables the INR to be obtained rapidly. POCT was employed in our pediatric patient to monitor INR because, during hospitalization, he did not cooperate with venous-blood collection and cried continuously. Fortunately, POCT requires only a spot of blood from a fingertip, which aided compliance. Our pediatric patient lived far from our hospital so we taught his mother to use the portable analytical instrument correctly, and the patient cooperated well.

**Figure 4:**
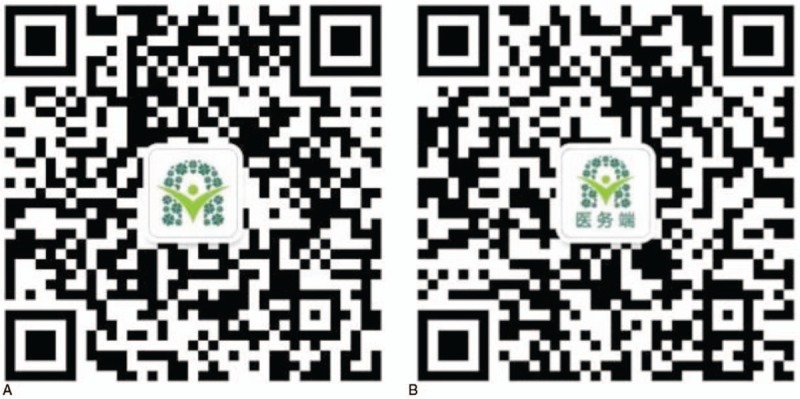
Anticoagulation Management app. A. Patient side. B. Medical side.

If this pediatric patient returned to our anticoagulation clinic for warfarin management, it would cost (in US dollars) 88 for transportation and 44 for a hotel stay; and the journey time would be 10 hours each way. If our pediatric patient joined the OAC, he could use POCT at home to obtain the INR, and the results would be sent to the OAC mobile-phone application (app) or the WeChat app (Fig. 4). Meanwhile, the pharmacist would check all the patient's information and respond to any adjustment to the warfarin dose and the time the next INR should be obtained.

His time within therapeutic range was 89.6% during 6 months after hospital discharge, much higher than that reported for other studies on anticoagulation management: 44%^[12]^ and 67%.^[13]^ Adverse reactions such as bleeding or thrombosis did not occur.

## 3 Discussion

In this paper, we use a warfarin decision support tool. WDC was transferred from a population PKPD-model for warfarin developed in NONMEM to a platform independent tool written in Java. The tool proved capable of solving a system of differential equations that represent the pharmacokinetics and pharmacodynamics of warfarin with a performance comparable to NONMEM. To estimate an a priori dose the user enters information on body weight, age, baseline and target INR, and optionally CYP2C9 and VKORC1 genotype. By adding information about previous doses and INR observations, the tool will suggest a new dose a posteriori through Bayesian forecasting. Results are displayed as the predicted dose per day and per week, and graphically as the predicted INR curve. The tool can also be used to predict INR following any given dose regimen.^[11]^

Our patient was the youngest infant to have a heart-valve replacement in our hospital for 158 years. We have little experience with anticoagulation therapy in children, and are continuing to explore it.

The warfarin dose for this infant was predicted using different models. The initial dose was 0.62 mg/day and maintenance dose was 0.98 mg/day using the WDC. The maintenance dose calculated using the Biss model was 1.057 mg/day.^[14]^ The predicted dose using the IWPC model was 3 mg/day. Guidelines set by the American College of Clinical Pharmacy (9th edition) recommended a dose for pediatric patients of 1.7 mg/day. Neither the Gage model nor FDA recommendations could be used to predict the dose for pediatric patients. When our patient reached the target INR, the warfarin dose was 0.94 mg/day, which was similar to the predicted maintenance dose using the WDC and Biss model. Therefore, this case illustrated that Hamberg and Biss models were equally applicable to this Chinese pediatric patient.

At present, multivariable linear regression (MLR) and maximum a posteriori probability Bayesian (MAPB) estimation methods are used in the formulation of individualized regimens for warfarin. The method for the MLR calculation is relatively simple and the warfarin dose can be estimated according to the pathophysiology indices and genotypes of the patient.^[8]^ The MAPB method must be fitted with the compartmental model, pharmacokinetic parameters and parameter distribution estimated, and then the patient genotype and previous INR values must be combined to obtain more complete individual pharmacokinetics/pharmacodynamics parameters. Finally, according to the baseline INR, target INR, age, weight and genotype, the warfarin maintenance dose can be predicted. The MAPB method can be used to predict the INR based on age, body weight, warfarin dose, target INR, baseline INR, interval of administration, and frequency of administration.^[11]^ The Biss model was constructed using the MLR method, so it can predict only the warfarin dose; it cannot predict the INR and cannot use previous INR results to predict the maintenance dose of warfarin again. The WDC was constructed based on the MAPB method, so it can predict the initial dose of warfarin but also the maintenance dose of warfarin by using previous INR results, and also can predict the INR.^[14]^

Infants with large body surface distribution and faster drug clearance rate,^[15]^ therefore, FDA instructions indicate that the warfarin dose should be adjusted constantly with changes in age, medication and diet for infants. Frequent monitoring of the INR is recommended.^[7]^ POCT can detect the INR in just a few minutes by collecting blood from fingertip capillaries. POCT is suitable for children. Pediatric patients can contact anticoagulant pharmacists through the OAC at home to improve the safety and efficacy of anticoagulation treatment by adjusting the dose in a timely manner. In addition, POCT can improve the satisfaction/compliance of patients and efficacy of anticoagulation therapy, reduce the risks of thromboembolic events and mortality, and does not increase the risk of bleeding events.^[16]^ For our patient, POCT was suitable for anticoagulation management using the OAC.

## Author contributions

**Conceptualization:** Hua Cao, Jinhua Zhang.

**Data curation:** Xiaotong Xia, Xuan Xia, Jinhua Zhang.

**Formal analysis:** Jinglan Fu, Ying Dai, Jinhua Zhang.

**Project administration:** Hua Cao.

**Writing – original draft:** Xiaotong Xia, Jinhua Zhang.
